# Watch Me Play!: protocol for a feasibility study of a remotely delivered intervention to promote mental health resilience for children (ages 0–8) across UK early years and children’s services

**DOI:** 10.1186/s40814-024-01491-7

**Published:** 2024-04-04

**Authors:** Elizabeth Randell, Claire Nollett, Josie Henley, Kim Smallman, Sean Johnson, Lena Meister, Rachel McNamara, David Wilkins, Jeremy Segrott, Angela Casbard, Jenifer Wakelyn, Kathy McKay, Ekaterina Bordea, Vaso Totsika, Eilis Kennedy

**Affiliations:** 1 Centre for Trials Research, Cardiff University, Cardiff, Wales; 2 School of Social Sciences, Cardiff University, Cardiff, Wales; 3 Centre for Trials Research, DECIPHer Centre, Cardiff University, Cardiff, Wales; 4 Tavistock and Portman NHS Foundation Trust, London, England; 5 University College London, London, England

**Keywords:** Parent–child interaction, Child development, Communication

## Abstract

**Background:**

Half of mental health problems are established by the age of 14 years and 75% by 24 years. Early intervention and prevention of mental ill health are therefore vitally important. However, increased demand over recent years has meant that access to child mental health services is often restricted to those in severest need. Watch Me Play! (WMP) is an early intervention designed to support caregiver attunement and attention to the child to promote social-emotional well-being and thereby mental health resilience. Originally developed in the context of a local authority mental health service for children in care, it is now also delivered online as a low intensity, scalable, preventative intervention. Although WMP shows promise and is already used in some services, we do not yet know whether it is effective.

**Methods:**

A non-randomised single group feasibility study with embedded process evaluation. We propose to recruit up to 40 parents/carers of children aged 0–8 years who have been referred to early years and children’s services in the UK. WMP involves a parent watching the child play and talking to their child about their play (or for babies, observing and following signals) for up to 20 min per session. Some sessions are facilitated by a trained practitioner who provides prompts where necessary, gives feedback, and discusses the child’s play with the caregiver. Services will offer five facilitated sessions, and parents will be asked to do at least 10 additional sessions on their own with their child in a 5-week period. Feasibility outcomes examined are as follows: (i) recruitment, (ii) retention, (iii) adherence, (iv) fidelity of delivery, (v) barriers and facilitators of participation, (vi) intervention acceptability, (vii) description of usual care, and (viii) data collection procedures. Intervention mechanisms will be examined through qualitative interview data. Economic evaluation will be conducted estimating cost of the intervention and cost of service use for child and parents/carers quality-adjusted life years.

**Discussion:**

This study will address feasibility questions associated with progression to a future randomised trial of WMP.

**Trial registration:**

ISRCTN13644899. Registered on 14th April 2023.

## Background

Public Health England report that half of mental health problems are established by the age of 14 years and 75% by 24 years [1]. Early intervention and prevention of mental ill health are therefore vitally important. However, increased demand over recent years has meant that access to child mental health services is often restricted to those in severest need. In 2019–2020, only a quarter of children estimated to need help received it [2], and difficulties accessing treatment remained a key concern in 2021 [3, 4]. Those not offered help include children at higher risk of developing problems later and those with problems that do not meet service thresholds [5]. Important opportunities for prevention and treatment are therefore missed, and resource-stretched services and practitioners are left frustrated at not being able to intervene at an optimal time [6].

Children in care — children who are under the care of a local authority (child welfare system) — are known to be at high risk of developing mental health problems in childhood and adolescence [7–9]. Watch Me Play! (WMP) was originally developed in the context of a local authority mental health service for children in care to offer an intervention to babies and children who would otherwise be offered little. It is an early intervention designed to support caregiver attunement and attention to the child to promote child social-emotional well-being and thereby mental health resilience. WMP can be delivered in the first weeks of a baby’s life up to the age of 8 years depending on the kind of play they enjoy and are ready for. WMP involves a parent or carer watching the child play freely, while the parent talks with their child about their play for a period of up to 20 min (this is called one session). The parents prepare by switching off the TV, phone, and screens and putting out a small selection of nonelectronic toys. The parent watches their child as they play, only joining in if the child invites them to do so, allowing the child to lead the play, as long as this is safe. The parent follows the child’s play and describes what the child is doing. The same ideas apply in WMP with babies before they are of an age to play with toys: the parent notices and follows the baby’s signals; mirrors facial expressions, movements, and sounds; and talks to the baby, imitating the baby’s expressions or sounds as if having a conversation. This can give an idea of what the baby is interested in. The parent does not direct the child’s play. The parent does not engage in other activities, giving instead their full attention to the child or baby during the 5- to 20-min session. The parents are encouraged to record their reflections in a diary at the end of a session. Some sessions are facilitated by a trained practitioner who joins the parent in watching the child or baby, either in person or online (using secure video conferencing software), talking to the child about their play, and providing prompts to the parent where necessary. Towards the end of the session, the trained practitioner discusses the child’s play with the parent: what they saw, what was new or not new, and what the child enjoyed or was frustrated by and about the parent’s experience: what they noticed, enjoyed, or found difficult. A facilitated session with a practitioner lasts up to 1 h. WMP is a flexible model that fits in with parents’ time availability, the needs of the family, and the resources that services can offer.

The first manual of WMP was published in 2019, followed by a revised and expanded version in 2020 [10]. Since its publication, demand for the intervention has surged with services wanting to introduce it and practitioners asking for training. Practitioners interested in WMP come from a range of health, education, and social care services, as WMP is an intervention that can support families in different contexts. The publication of the manual in 2020 coincided with COVID-19 lockdowns when many services supported their clients or patients remotely. Therefore, in 2020 and 2021, WMP was delivered online or in combination of online and face-to-face sessions by services. Early years and children’s services are accepting referrals for families and children faced with various challenges, such as parent–child relationship difficulties, suspected infant mental health problems, parent mental health problems, and child developmental delay.

WMP therefore has the potential to address the need for a low intensity, scalable, preventative intervention, inclusive of a broad age range (0 to 8 years) that can be offered by practitioners in NHS, local authority, and voluntary sector settings. It has the potential to address key challenges for children’s mental health identified in the 2021 Children’s Commissioner for England’s report of both increasing access to intervention for children and broadening the ‘system of support’ on offer across a range of services [2]. This study directly addresses priority 4 of the top 10 priorities for children’s mental health identified by the James Lind Alliance, i.e. ‘What are the most effective early interventions or early intervention strategies for supporting children and young people to improve mental resilience?’ [11]. The key importance of early intervention in improving children’s lifelong mental health is further highlighted in the 2021 DHSC Early Years Health and Development Review Report: ‘The Best Start for Life: A Vision for the 1,001 Critical Days’ [12]. Maximising opportunities for prevention and improving access are also noted as priorities in the Framework for Mental Health Research [13] and in the Mental Health Research Goals 2020–2030 [14].

As a preventative intervention, WMP is designed to complement or precede other interventions, e.g. video-feedback or parent-infant therapy. WMP is not resource-intensive to deliver, which may enable services to increase access and address barriers to engagement that may limit the need for more intensive approaches. WMP also addresses a wide population, which may be of benefit in circumstances where there are additional barriers to accessing mental health support, e.g. children in the care system, remote rural areas, areas of high deprivation, and ethnic minority communities. The broad age range (0–8 years) includes the possibility of early intervention in infancy when relationships and developmental trajectories may be most amenable to change. It is therefore essential that the evidence base for WMP is developed to enable services to offer the right support to families. The first step in this process is to formally assess the feasibility and acceptability of WMP for families referred to early years and children’s services, either currently experiencing mental health problems or at significant risk of developing mental health problems in later life. Results of the present study will indicate whether it is feasible and appropriate to conduct a further evaluation of WMP.

## Methods

### Objectives

The primary objective is to determine the feasibility of delivering WMP to families of young children (aged 0 to 8 years) referred to early years and children’s services in the UK.

To achieve the primary objective, the following will be assessed:

1. The feasibility of recruiting families, recruitment rates, adherence to the intervention, and retention rates (the number of families remaining in the study at 3 months)
2. The feasibility of recruiting and training suitable intervention providers and facilitators to deliver the WMP intervention
3. Implementation of WMP (online and face to face)
4. The acceptability of study processes to delivery organisations, delivery staff, and parents/carers
5. The acceptability, barriers, and facilitators of the WMP intervention to delivery organisations, delivery staff, and parents/carers to inform a future trial
6. Intervention receipt and hypothesised mechanisms of action in order to refine the intervention logic model
7. Intervention costs and the feasibility of conducting a full economic evaluation in a future trial
8. Treatment as usual (TAU) as delivered by participating services, how WMP interacts with or is delivered in relation to TAU, and the most appropriate comparator for a definitive trial
9. A primary outcome for a future trial

### Study design and setting

A non-randomised single group feasibility study, including a process evaluation. Participants will be recruited from early years, children’s health services and some social care, education, or voluntary services. These are services designed to support families of young children. The study will be carried out at sites spread across the UK serving a mix of populations. Sites may include a mixture of CAMHS, child development teams, foster care services, and mental health services for children in care. We will offer to train up to three members of staff in WMP so that each service has at least three staff trained. We will aim to recruit some sites with no prior exposure to WMP so we can investigate the feasibility of rolling out training.

### Inclusion criteria

Parents or carers of children aged 0 to 8 years old who have a referral to or have been accepted by an early years/children’s service within the United Kingdom (UK). Parents must be able to complete outcome measures in English (with support if required, whereby a researcher can talk through the questionnaire via telephone). Parents/carers of children with any type of mental health problem, and/or developmental delay, will not be excluded from taking part provided all eligibility criteria are met.

### Exclusion criteria

Other than the obverse of the inclusion criteria, participants will be excluded if as follows: parents/carers are currently receiving or planning to receive WMP not within the context of this study in the following 6 months.

### Intervention

**Fig. 1:**
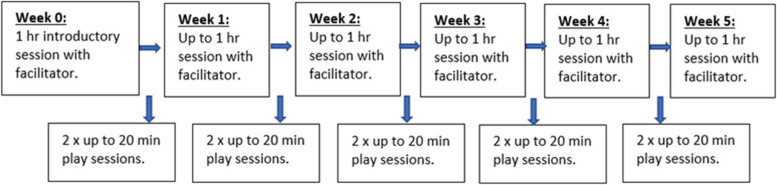
Intervention flow diagram

In this study, it is recommended that services offer 5 facilitated sessions, following an introductory meeting, and parents do at least 10 independent sessions with their child over a 5-week period (i.e. three sessions a week, of which one is facilitated) (Fig. 1).

We will be monitoring fsrequency of sessions to see what works for families. As mentioned above, since WMP’s publication in 2020, families have been supported in face-to-face sessions as well as remotely, with practitioners supporting parents through Zoom or Teams during the facilitated sessions. In this study, WMP will be primarily delivered online, but where the parent or the WMP practitioner feel that some in-person contact is important, services may offer face-to-face facilitated sessions (e.g. the introductory meeting and/or one facilitated session). Many services have returned to face-to-face contact, but we want to see how WMP could provide a flexible model of support through a mixture of face-to-face and online sessions. This study will monitor the format of facilitated sessions across all participating sites.

A parent and therapist WMP manual [10] and leaflets in different languages are available for free download from this webpage: https://tavistockandportman.nhs.uk/watch-me-play. Background information, a video illustrating the approach, and further materials for parents and for practitioners are available from www.watchmeplay.info.

Any healthcare, social care, or early years professional with 2 or more years’ experience of working with children and families can be trained in the approach. Training is a 1-day workshop introduction followed by group supervision.

The manual and other resources are intended to help parents to support their child’s development through play and cover the following:

1. What is Watch Me Play!
2. How to do Watch Me Play! — Quick View
  - Preparing
  - Baby and child-led play
  - Watching your child play
  - Talking with your baby or child about their play
  - Talking with another adult about the child’s play
3. Toys and materials for play
4. A Watch Me Play! Diary
5. Why play matters

Two shorter leaflets and a 4-min video explaining the approach are also available for parents.

Supervision: Four group supervision meetings will take place during the 5-week intervention period. Group supervision involves WMP practitioners taking turns to discuss their case with the WMP supervisor drawing on written notes of a recent session. An additional option of a monthly drop-in supervision will be available to discuss issues arising.

As part of regular practice, practitioners complete a 5-item WMP checklist after each session with caregiver(s). Each of the 5 items is rated as ‘achieved’ (2), ‘partially achieved’ (1), ‘not yet achieved’ (0), and ‘explored with caregiver?’ (yes = 1, no = 0). For a session to be completed with acceptable fidelity, a score of 10 out of 15 is expected.

### Outcomes

As the current study is not a trial, no formal progression criteria will be established. Feasibility outcomes will be examined across the board to determine the design of the next study. This is a mixed-methods approach, and the following outcomes will be measured (see the ‘Qualitative analysis’ section for further definition):

1. Recruitment
2. Retention
3. Adherence to the intervention
4. Fidelity of WMP programme delivery
5. Assessment of the barriers and facilitators to implementation and variation across context (online or face to face)
6. Acceptability of WMP to parents, WMP practitioners, and service managers
7. Treatment-as-usual (TAU) description
8. Acceptability and feasibility of data collection procedures

In addition to feasibility outcomes, intervention mechanisms will be examined through qualitative data from interviews with parent/carers and delivery staff on how they experienced the process of WMP and perceived impacts.

As part of testing the feasibility of evaluation procedures, child/parent outcome and health economic measures (detailed in Table 1 below) will also be used. Child/parent outcomes were selected for inclusion in this study on the basis of WMP’s logic model (theory of change).

**Table 1:** Parent/carer-reported outcomes and health economic measures

Construct	Measure
Child mental health	Child Behavior Checklist (CBCL) [15]
	Strengths and Difficulties Questionnaire (SDQ) [16]
Child socialisation and communication	Vineland Adaptive Behavior Scale 3 (VABS) [17]
Parenting stress	Parent Stress Index-Short Form (PSI) [18, 19]
Parenting competence	Being a Parent Scale [20]
Parent–child relationship quality	Mothers’ Object Relations Scale-Short Form (MORS-SF) and MORS (child) for 0–4 years [21, 22], Parent–Child Relationship Scale for 5 + years [23], and the frequency of parent–child activities (Parent–Child Activity Index [24])
Parent–child interaction	A 20-min videotaped-free play interaction between the parent/caregiver and the child (*n* = 8 baseline participants)
Parent/carer health-related quality of life and quality-adjusted life years	EQ-5D-5L [25]
Service use for child	A modified version of the Child and Adolescent Service Use Schedule (CA-SUS) [26]

The child’s developmental delay status will be recorded at baseline through parent self-report along with demographic information and information as to the child’s status as in contact with social worker.

### Sample size

The study aims to recruit up to 40 families (1 caregiver of 1 child) from across up to 15 sites. As this is a feasibility study, a power calculation was not utilised to estimate the target sample size. Instead, we looked to the literature to guide us in deciding an appropriate recruitment target.

### Recruitment

Participants will be identified by services as potentially eligible: we will ask the direct care team to identify children within the right age range and in particular those with a social worker either currently or in the past 24 months. Members of the direct care team will email parents/carers a brief information leaflet (briefing sheet) about the study. Parents interested in taking part or knowing more will either go straight to the screening/consent questionnaire via a link code on the briefing sheet or contact the research team via contact details on the briefing sheet. The researcher will discuss the study in more detail with the participants. The screening/consent questionnaire will contain the full participant information sheet. Those who provide informed consent will be screened for eligibility. We will proactively recruit parents/carers of children with or who have had a social worker, noting barriers and facilitators to recruitment where they exist. Recruitment will be monitored to ensure diversity in relation to ethnicity, low SES, and poor literacy, as per NIHR INCLUDE and INCLUDE ethnicity guidance [27, 28]. We will encourage BME participation following guidance from the Centre for BME health (https://centreforbmehealth.org.uk/).

A £50 voucher at baseline and follow-up will be offered to families taking part. A £50 voucher will also be offered to participants taking part in qualitative interviews and those who take part in the free play video recording. To address digital poverty and support the inclusion of those who might struggle to participate, we will offer £20 to support those who may not have adequate Internet data to undertake the online evaluation.

### Data collection

Outcomes will be measured at baseline and post intervention. All outcomes, except for parent–child interaction, will be measured through parent report (questionnaires to be completed via a survey link). Parent–child interaction during free play will be video recorded (for 20 min) by researchers remotely during baseline only for a smaller number of randomly selected participants, from those who have consented to be contacted (up to *n* = 8). The purpose is to assess the feasibility and acceptability of collecting data in this way in a future trial. Additional information will be collected at screening only on the presence of developmental delay and on the child’s status as in contact with social worker.

In the event that participants’ follow-up data collection appointments are missed at the proposed time points, the research team will contact the participant by telephone to rearrange the appointment as soon as possible. Follow-up assessments for all participants will be conducted 3 months from baseline with a + / − 2-week window.

### Process evaluation

The process evaluation will examine (i) recruitment and reach, (ii) retention (iii) engagement and adherence, (iv) intervention implementation, and (v) intervention acceptability, barriers, and facilitators of participation. We will use MRC guidance for process evaluation of complex interventions [29] as a framework to describe implementation processes, examine intervention mechanisms, and consider how the intervention interacts with existing delivery systems across different contexts (e.g. urban/rural areas). A mixed-methods approach will be used. Quantitative methods will assess recruitment rates/patterns and intervention fidelity/adherence. Qualitative interviews with participants and delivery staff, including trainers, will examine implementation processes, intervention mechanisms, and the role of contextual factors.

### Qualitative data collection

Semi-structured interviews will be conducted virtually or by telephone. Topic guides will be developed using a scoping literature review and input from the research team and PPI advisory panel. Interviews with parents will explore their experience of receiving the intervention, including perceived benefits and mechanisms. Interviews will also be conducted with staff members who have delivered the intervention. We may invite other staff members to interview, e.g. managers in the organisation who have been involved but have not delivered the intervention.

The number of interviews will be based on preliminary analysis/interviewer field notes indicating whether the data collected sufficiently answer the research questions. Our proposed sample size for interviews with parents is up to 20 and for staff is 6–8.

### Data management

All questionnaire data will be collected using electronic data capture. This will be through a web-based survey designed specifically for this trial using Qualtrics and through Q-Global and PARiConnect for the VABS and PSI respectively. All outcome measures are completed by participants at baseline and upon completion of the intervention, which will be approximately 3-month post-baseline.

### Progression

As a feasibility study evaluating WMP for the first time, no predefined progression criteria have been set. This study will include a comprehensive assessment of multiple factors to inform decision-making about the viability of a future evaluation. This will include qualitative assessments alongside quantitative analysis of feasibility outcomes with a particular emphasis on recruitment and intervention completion for the study period. We will consider these alongside factors such as stakeholder perceptions, regulatory environment, and recruitment landscape.

### Statistical methods

The primary outcome is to determine the feasibility of future research. This is a mixed-methods approach, including qualitative interviews. The quantitative measures contributing to the primary feasibility outcomes will be as follows:

1. Recruitment feasibility: The number of families invited to take part and the number and percentage who attend at least one WMP session
  - Number and percentage of children with reported developmental delay recruited
  - Number and percentage of children with contact with a social worker recruited
2. Retention: The number and percentage of families who remain on the study at 3 months
3. Adherence is as follows:
  - Number of online and/or face-to-face WMP sessions.
  - Number of independent and/or facilitated WMP sessions.
  - The number and percentage of families who completed 10/15 sessions including all 5 facilitated sessions.
4. Fidelity of programme delivery: Quantitative data from the standardised WMP checklist will be descriptively summarised with tabulations and graphics. Practitioners will complete a short WMP checklist after each session with caregiver(s). Checklists will be rated according to fidelity criteria to determine whether acceptable fidelity has been achieved.
5. Acceptability for families is as follows:
  - The number of questionnaires (EQ-5D-5L and service use) completed by families at each time point.
  - Number of remote videos captured by families completing this element and number of videos over 5-min duration

Outcome measures related to the clinical, quality of life, and health economics are as follows:

1. Cost of WMP: Total costs attributed to WMP from study sites and cost per child.
2. Identification of potential outcomes and assessments for a future trial:
  - Descriptive tabulations of baseline demographic information including social worker contact status at baseline.
  - Descriptive tabulations and graphics showing responses to the parent/child and health economic measures listed in Table 1.
  - Number and percentage of children in the programme with existing mental health problems (as defined by the clinical cut-off score of the CBCL) at baseline, number, and percentage of children with sustained, improved, and worsened mental health problems at 3 months, as reported by the families, according to changes in CBCL scores from baseline.

Descriptive analysis will summarise data for all participants and will include tabulations of categorical data, with the median and interquartile ranges of quantitative measures (such as questionnaire scales). Where percentages are calculated, these will be presented with 95% confidence intervals. Graphics will be used for some outputs to show how data vary over time. Demographic, medical history, and baseline data will be summarised, and then the interventional sessions will be summarised for each session. The 3-month timepoint will be the follow-up timepoint for determining outcomes for a future trial.

There are no formal statistical tests required for this analysis.

### Qualitative analysis

Interview transcripts will be analysed using thematic analysis [30]. After familiarisation of data, we will generate preliminary codes to label data of interest based on the research objectives. We will retrieve coded data to generate themes and produce summaries of interviewees’ talk on each theme, for each individual participant, and visually arrange it in a table to build an overall picture of the whole data set. This will allow for comparison across parents/carers, staff, and sites to identify variation and similarities in the final stage of interpretation of data. The next stage will involve the research team using the summaries to examine the quality and boundaries of themes identified. From this, we will finalise a thematic map refining the specifics of each theme to capture key concepts and produce analytical commentary and interpretation of the data set as a whole and connect with the original research objectives. The qualitative software package, NVivo (2015), will be used to manage the data. A proportion of transcripts will be double-coded until consensus is reached (likely to be 10%).

We will use the qualitative data to explore the perspectives of parents/carers and staff and to explore acceptability of delivering the intervention (staff) and to identify barriers and facilitators (practical, management, organisational) to implementing virtual or hybrid WMP in other sites which do not routinely use a virtual delivery.

### Economic evaluation

We will assess the costs of delivering facilitated WMP and report a cost per child of delivering WMP, including training cost and time spent on different components of the intervention.

The feasibility of collecting service use data and health-related quality-of-life (HRQL) information will also be evaluated for use in a future cost-effectiveness analysis. Service use will be collected using a modified version of the Child and Adolescent Service Use Schedule (CA-SUS) at baseline and 3 months after the intervention. We will report information on levels of service use and completeness to assess the feasibility of using the questionnaire and whether any changes should be made for a future study. We will report the mean total cost of service use per child. We will collect the self-reported EQ-5D-5L from parents/carers to assess their HRQL at baseline and 3 months after the intervention. We will calculate index values of the UK value set [31] and calculate quality-adjusted life years (QALYs) using area under the curve approach. We will report completeness, mean index value at each timepoint, and QALYs over the course of the study.

### Oversight and monitoring

A Study Management Group (SMG) will include the chief investigators, co-applicants, collaborators, trial manager, data manager, health economist, statistician, and administrator. The SMG will meet approximately every 4–6 weeks throughout the course of the study. Members will be required to sign up to the remit and conditions as set out in the SMG Charter.

An Executive Committee (EC) has been set up to provide independent oversight. It comprises of an independent chair, one local PI responsible for a (likely) participating site (who brings experience of WMP implementation and study participation), one independent WMP expert (research in families and children), and one member of the Parent Carer Advisory Group. The EC will meet at least three times during the course of the study to provide overall supervision for the trial and provide advice through its independent chair. EC members will be required to sign up to the remit and conditions as set out in the EC Charter. Given the low-risk nature of the trial, we will ask the EC to act as Data Monitoring Committee (DMC).

### Adverse event reporting and harms

All SAEs must be reported immediately (and within 24 h of knowledge of the event) by the principal investigator at the participating site to the study team unless the SAE is specified as not requiring immediate reporting. In addition, for the purposes of this study, the removal of a child from the biological family (or unplanned removal more specifically) is considered to be an adverse event, and any instances will be recorded.

### Data protection

Data will be stored confidentially on secure password-protected severs and accordance with the Data Protection Act 2018 and General Data Protection Regulation (GDPR). Personal information will be collected, kept, and stored securely in compliance with UK GDPR. The research team and staff at participating sites are trained in GDPR compliance. The data controller is the Tavistock and Portman NHS Foundation Trust. The data custodian for this study is the CI, Dr. Eilis Kennedy. A Data Protection Impact Assessment (DPIA) has been completed as part of an overall trial risk assessment.

## Discussion

The proposed feasibility study will address feasibility and acceptability questions associated with delivery of Watch Me Play! for families referred to early years and children’s services, either currently experiencing mental health problems or at significant risk of developing mental health problems in later life. This is the first evaluation of WMP and the first evaluation with this population. Results of this study will indicate whether it is appropriate to conduct further evaluations of WMP, including whether a randomised feasibility evaluation with formal progression criteria is warranted.

### Study status

This manuscript has been drafted according to Version 4.0 (11th January 2024) of the trial protocol. The final report will follow the CONSORT (Consolidated Standards of Reporting Trials) statement. The study is sponsored by the Tavistock and Portman NHS Foundation Trust (sponsor.noclor@nhs.net).

## Data Availability

The datasets used and/or analysed during the current study are available from the corresponding author on reasonable request.

## Abbreviations

CTR: Centre for Trials Research
CI: Chief investigator
GDPR: General Data Protection Regulation
UK: United Kingdom
DMC: Data Monitoring Committee
MRC: Medical Research Council
RCT: Randomised controlled trial
DPIA: Data Protection Impact Assessment
SAE: Serious adverse event
SMG: Study Management Group
EC: Executive Committee
WMP: Watch Me Play!
CONSORT: Consolidated Standards of Reporting Trials

## References

[1] Public Health England. Universal approaches to improving children and young people’s mental health and wellbeing: report of the findings of a Special Interest Group. [Internet]. 2019 [cited 2021 Sep 21]. Available from: https://assets.publishing.service.gov.uk/government/uploads/system/uploads/attachment_data/file/842176/SIG_report.pdf.

[2] Children’s Commissioner. The state of children’s mental health services 2020–21 [Internet]. 2021 [cited 2021 Sep 21]. Available from: https://www.childrenscommissioner.gov.uk/wp-content/uploads/2021/01/cco-the-state-of-childrens-mental-health-services-2020-21.pdf.

[3] BBC. Child mental health waiting times ‘deeply disturbing’ [Internet]. 2021 [cited 2021 Sep 22]. Available from: https://www.bbc.co.uk/news/uk-scotland-56257753.

[4] BBC. Children face ‘agonising’ waits for mental health care [Internet]. 2021 [cited 2021 Sep 22]. Available from: https://www.bbc.co.uk/news/health-58565067.

[5] Crenna-Jennings W, Hutchinson J. Access to children and young people’s mental health services [Internet]. 2020 [cited 2021 Sep 21]. Available from: https://epi.org.uk/publications-and-research/access-to-child-and-adolescent-mental-health-services-in-2019/.

[6] Colizzi M, Lasalvia A, Ruggeri M (2020). Prevention and early intervention in youth mental health: is it time for a multidisciplinary and trans-diagnostic model for care?. Int J Ment Health Syst.

[7] National Youth Advocacy Service. Looked after minds: prioritising the mental health of care-experienced children and young people. 2019 [cited 2021 Sep 17]; Available from: https://www.nyas.net/wp-content/uploads/NYAS-looked-after-mind-report.pdf.

[8] York W, Jones J (2017). Addressing the mental health needs of looked after children in foster care: the experiences of foster carers. J Psychiatr Ment Health Nurs.

[9] Care Leaver Covenant. Care leaver covenant [Internet]. 2018 [cited 2021 Nov 2]. Available from: https://mycovenant.org.uk/wp-content/uploads/2020/01/CLC-Intro-Leaflet-Branded-Low.pdf.

[10] Wakelyn J, Katz A. Watch Me Play! Manual for parents, Version 2 [Internet]. Tavistock and Portman NHS Foundation Trust; 2020 [cited 2021 Sep 21]. Available from: https://tavistockandportman.nhs.uk/watch-me-play.

[11] McPin Foundation. Research priorities for children and young people’s mental health: interventions and services [Internet]. 2018 [cited 2021 Sep 22]. Available from: https://www.jla.nihr.ac.uk/priority-setting-partnerships/Mental-health-in-children-and-young-people/downloads/Mental-Health-in-Children-and-Young-People-PSP-Main-Report.pdf.

[12] Department of Health and Social Care. The best start for life: a vision for the 1,001 critical days [Internet]. 2021 [cited 2021 Sep 22]. Available from: https://www.gov.uk/government/publications/the-best-start-for-life-a-vision-for-the-1001-critical-days.

[13] Department of Health and Social Care. A framework for mental health research. 2017.

[14] Academy of Medical Sciences. Mental health research goals 2020–2030 [Internet]. 2020 [cited 2021 Sep 22]. Available from: https://acmedsci.ac.uk/file-download/63608018.

[15] Achenbach TM. Child Behavior Checklist. In: Encyclopedia of Clinical Neuropsychology. New York, NY: Springer New York; 2011. p. 546–52.

[16] Goodman R (2001). Psychometric properties of the Strengths and Difficulties Questionnaire. J Am Acad Child Adolesc Psychiatry.

[17] Sparrow S, Cicchetti D. Vineland adaptive behaviour scales [Internet]. 2016 [cited 2021 Sep 21]. Available from: https://www.pearsonclinical.co.uk/psychology/childmentalhealth/childadaptivebehaviour/vineland-3/vineland-adaptive-behavior-scales-third-edition-vineland-3.aspx.

[18] Abidin R, Flens JR, Austin WG, Archer RP (2006). The Parenting Stress Index. Forensic uses of clinical assessment instruments.

[19] Haskett ME, Ahern LS, Ward CS, Allaire JC (2006). Factor structure and validity of the Parenting Stress Index-Short Form. J Clin Child Adolesc Psychol.

[20] Johnston C, Mash EJ (1989). A measure of parenting satisfaction and efficacy. J Clin Child Psychol.

[21] Oates J, Gervai J, Danis I, Lakatos K, Davies J (2018). Validation of the Mothers’ Object Relations Scales Short-Form (MORS-SF). J Prenat Perinat Psychol Heal.

[22] Simkiss DE, MacCallum F, Fan EE, Oates JM, Kimani PK, Stewart-Brown S (2013). Validation of the mothers object relations scales in 2–4 year old children and comparison with the child–parent relationship scale. Health Qual Life Outcomes.

[23] Driscoll K, Pianta RC (2011). Mothers’ and fathers’ perceptions of conflict and closeness in parent-child relationships during early childhood. J Early Child Infant Psychol.

[24] Totsika V (2015). Child-parent activity index.

[25] Herdman M, Gudex C, Lloyd A, Janssen M, Kind P, Parkin D (2011). Development and preliminary testing of the new five-level version of EQ-5D (EQ-5D-5L). Qual Life Res.

[26] Byford S, Barrett B, Roberts C, Wilkinson P, Dubicka B, Kelvin RG (2007). Cost-effectiveness of selective serotonin reuptake inhibitors and routine specialist care with and without cognitive–behavioural therapy in adolescents with major depression. Br J Psychiatry.

[27] NIHR. Improving inclusion of under-served groups in clinical research: guidance from the NIHR INCLUDE project [Internet]. 2020 [cited 2021 Sep 21]. Available from: www.nihr.ac.uk/documents/improving-inclusion-of-under-served-groups-in-clinical-research-guidance-from-include-project/25435.

[28] Treweek S, Banister K, Bower P, Cotton S, Devane D, Gardner HR (2021). Developing the INCLUDE Ethnicity Framework—a tool to help trialists design trials that better reflect the communities they serve. Trials.

[29] Moore GF, Audrey S, Barker M, Bond L, Bonell C, Hardeman W, et al. Process evaluation of complex interventions: Medical Research Council guidance. BMJ. 2015;350(mar19 6):h1258–h1258. 10.1136/bmj.h1258 PMC4366184 25791983

[30] Braun V, Clarke V. Thematic analysis: a practical guide. QMiP Bull. 2022;1(33).

[31] Devlin NJ, Shah KK, Feng Y, Mulhern B, van Hout B (2018). Valuing health-related quality of life: an EQ-5D-5L value set for England. Health Econ.

